# Correction: Development of diagnostic SNP markers for quality assurance and control in sweetpotato [*Ipomoea batatas* (L.) Lam.] breeding programs

**DOI:** 10.1371/journal.pone.0233828

**Published:** 2020-05-21

**Authors:** The PLOS ONE Staff

## Notice of republication

An incorrect version of Fig 1 was published in error. The publisher apologizes for this error. This article was republished on May 5, 2020 to correct for this error. Please download this article again to view the correct version.
