# Supplementary figures and images for: Development of diagnostic SNP markers for quality assurance and control in sweetpotato [Ipomoea batatas (L.) Lam.] breeding programs
Source: PLoS One. 2020 Apr 24;15(4):e0232173. doi: 10.1371/journal.pone.0232173 (PMC7182229; doi:10.1371/journal.pone.0232173)

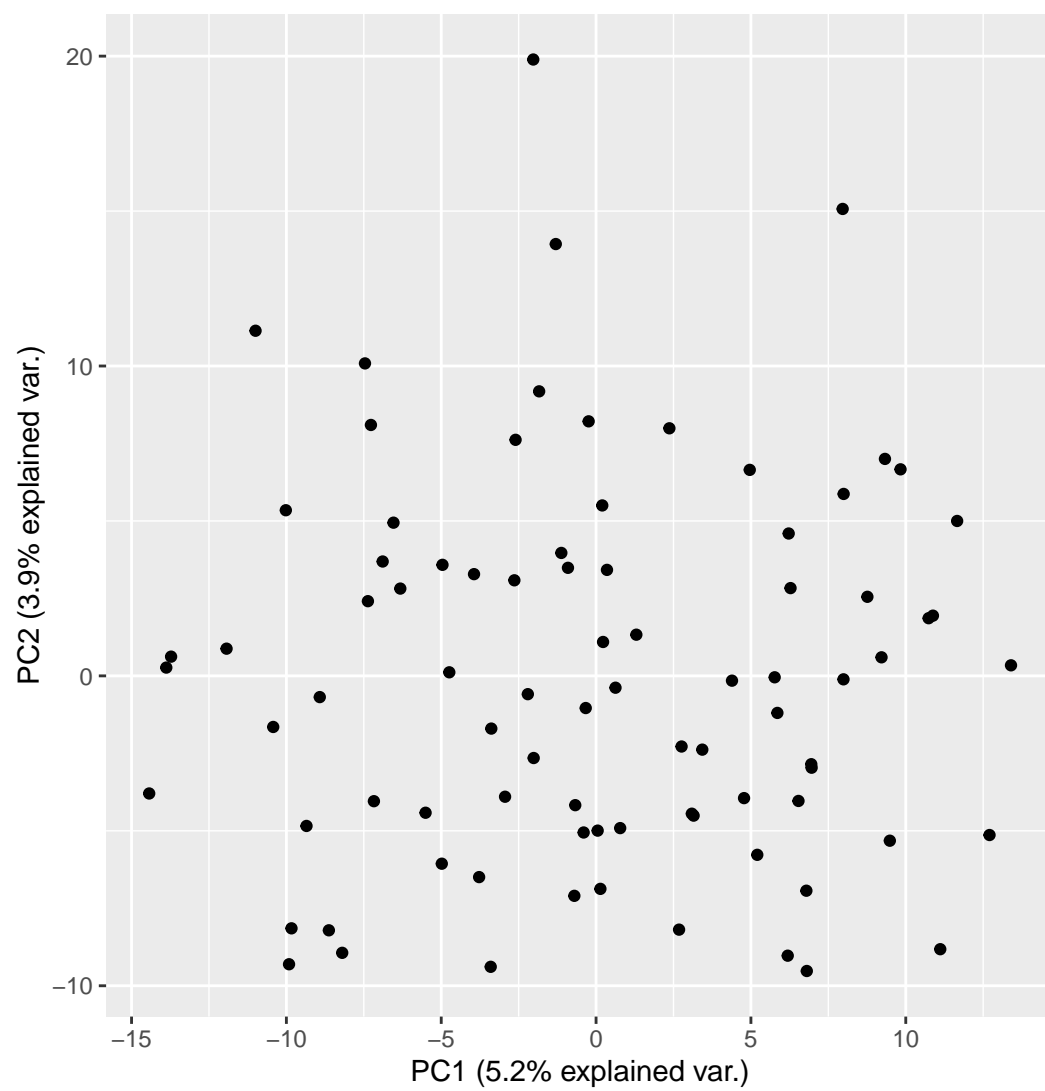

Supplement: S2 Fig — (PDF) [file pone.0232173.s005.pdf]
