# Supplementary material for: Development of diagnostic SNP markers for quality assurance and control in sweetpotato [Ipomoea batatas (L.) Lam.] breeding programs
Source: PLoS One. 2020 Apr 24;15(4):e0232173. doi: 10.1371/journal.pone.0232173 (PMC7182229; doi:10.1371/journal.pone.0232173)
Supplement: Attachment — Submitted filename: PONE-D-19-31393_reviewer_kim.docx [file pone.0232173.s009.docx]

I have a great need for submitted MS for sweet potato research.

I think the paper “Development of diagnostic SNP markers for quality assurance and control in sweetpotato [Ipomoea batatas (L.) Lam.] Breeding programs” is a good paper. However, I do not wish to print to PLOS ONE for the following reasons.

The development of the SNP marker, the subject of the study, is very difficult to apply in the hexaploid sweet potato. In addition, the scientists submitted the data with a lot of effort, but they did not present the phenotyping results decisively, so it could not be applied in a suitable breeding program.

I do not think there is any concern about double publishing, research ethics or publishing ethics.\

Kyung-Min Kim
